# Centering the role of community health workers in social risk screening, referral, and follow-up within the primary care setting

**DOI:** 10.1186/s12875-024-02590-3

**Published:** 2024-09-13

**Authors:** Emily K. Larson, Maia Ingram, Erin Dougherty, Maria Velasco, Vanessa Guzman, Azel Jackson, Kiran Patel, Scott C. Carvajal, Ada M. Wilkinson-Lee

**Affiliations:** 1 Arizona Prevention Research Center, Mel and Enid Zuckerman College of Public Health, University of Arizona, 1295 N Martin Ave, Tucson, AZ 85724 USA; 2 El Rio Health Center, 839 W. Congress Street, Tucson, Arizona, 85745 USA; 3 Valle Del Sol Community Health Center, 3877 N 7th St, Phoenix, AZ 85014 USA; 4 Department of Mexican American Studies, College of Social and Behavioral Sciences, University of Arizona, 1110 E. James Rogers Way, Tucson, AZ 85721 USA

**Keywords:** Community health worker (CHW), Federally Qualified Health Center (FQHC), Primary care, Community-based participatory research, Process map, Process evaluation, Social risk screening, Social determinants of health

## Abstract

**Background:**

Community health workers (CHWs) remain an underutilized resource in social risk diagnostics in the primary care setting. This process evaluation study seeks to assess the role of CHWs in social risk screening, referral, and follow-up through process mapping to identify barriers to the process for future quality improvement efforts.

**Methods:**

Researchers at the Arizona Prevention Research Center (AzPRC) engaged with two Federally Qualified Health Centers (FQHCs) in two of Arizona’s major urban areas to evaluate their internal processes for social risk screening and intervention. The Consolidated Framework for Implementation Research (CFIR) was used to direct a process mapping exercise to visually describe the workflow, gaps, and barriers to identifying and addressing social risk.

**Results:**

The process unveiled key areas for health system improvements in the community setting, the organizational setting, and in the implementation of social risk screening, referral, and follow-up. Further, process maps highlight the potential resources needed for effective CHW integration to address social risk in the primary care setting.

**Conclusions:**

Our findings demonstrate the importance of organizational tools, such as process mapping, to assist primary care settings in evaluating internal processes for quality improvement in addressing social risk and in effectively integrating the CHW workforce. Subsequent research will evaluate rates of social risk screening, referral, and follow-up within all of Arizona’s FQHCs and propose models for CHW integration to address social risk in primary care and strengthen social risk screening reach and effectiveness.

**Supplementary Information:**

The online version contains supplementary material available at 10.1186/s12875-024-02590-3.

## Background

The concept of social prescribing in the medical field is not a new one, yet it remains an understudied and underutilized tool for expanding a healthcare provider’s practice into the social areas that affect a patient’s health outcomes [1]. Social prescription is most commonly defined as the referral of patients in primary care to non-clinical, local services for social health and well-being, such as housing and nutrition [2]. Key to the success of social prescription is the inclusion of strategies to ensure that the appropriate social needs of the patient are identified, that there is a response to those needs through direct assistance or referral, and that there is follow up to ensure that the patient received related services to their satisfaction [3].

In 1997, the Bromley by Bow Centre in the United Kingdom (UK) developed the Health Living Centre, one of the first documented primary care offices using the model of social prescribing as a driving force for a healthy community [4]. This service was conceptualized as an approach to address health inequality by acknowledging the strong connection between socioeconomic factors and a person’s mental and physical well-being [5]. Behavioral health care settings have traditionally taken a more holistic approach to addressing social risk factors in direct relation to mental health, while it is only in the past two decades that attention to social determinants of health (SDOH) have become central in medical settings [6]. While there is growing attention to SDOH in health care settings, physicians are not well positioned to respond within the context of a medical visit. According to The Physician Foundation’s Part One of Three: 2022 Survey of America’s Physicians, 61% of physicians feel they have “little to no time and ability to effectively address their patient’s [social] determinants of health”, while 87% report a desire for greater time and ability to discuss such matters [7]. While these numbers alone are staggering, 83% of physicians report that trying to address a patient’s determinants of health contribute to the physician’s experience of burnout [7].

Acknowledging the role of social determinants of health (SDOH) in conversations around improving health equity is paramount, as an estimated 80% of health outcomes remain attributable to a person’s socioecological context [8] Given the important influence that issues such as housing and food access, for example, have on a patient’s health outcomes, it is of critical importance that public health bridges the relationship between primary care and a public health system-guided confrontation of social risk. Despite the desire of providers to have the capacity to address their patients social risks, other staff within the primary care setting may be better situated to take the time to address these needs [7]. Therefore, there is a growing need to develop effective systems-level approaches to identifying, integrating, and following up on social risks in patient care, which requires the commitment of several levels of staff in the clinical setting [8]. 

There is robust research documenting the positive impact of CHWs as members of healthcare teams on issues such as chronic disease risk and management [9–12]. However, their role in identifying and addressing social risk, as an integral part of primary care teams, is neglected in the literature. A key role for the CHW workforce is to create a bridge between community members and health and human services [13]. CHWs are highly-skilled leaders, who can naturally communicate with their community and draw on resources to address social needs [14]. While a surplus of evidence exists to indicate the strength of CHWs in addressing social risks by connecting patients to community resources, there remains a lack of literature on effective ways to integrate CHWs into primary care services for the purpose of social risk screening, referral, and follow-up to ensure that social risk is addressed [15–21]. Common issues around social risk identification and intervention as an organizational norm include unclear referral channels between healthcare providers and CHWs, not having dedicated time for patients to complete the screening, mounting and existing administrative burden, and concerns with identifying proper billing codes in the electronic health record (EHR) [15–21]. 

The objective of this process evaluation study is to assess the role of CHWs in screening, referral, and follow-up for social risk within two FQHCs in order to identify barriers to the process for future quality improvement efforts.

## Methods

Using a community-based participatory research (CBPR) approach, academic partners collaborated with staff of two FQHCs interested in evaluating organizational procedures for social risk and the role of CHWs from screening to follow-up. CBPR is best described as a research methodology, which combines knowledge and action to encourage social change [22]. CBPR is founded on the principle that community partners and academic researchers are equal collaborators [22]. This CBPR study grew out of a larger academic-community research partnership between several FQHCs and county health departments to develop a CHW-driven community-clinical linkage (CCL) model [23, 24]. The research study coincided with national efforts to increase social risk screenings in FQHCs, leading to additional questions about the internal social risk screening process for two of the partner FQHCs [25]. Both FQHCs expressed interest in evaluating the role of CHWs in the social risk screening, referral, and follow-up processes for future quality improvement efforts.

Each author on this paper had a key role in the elaboration of this study. The following describes the roles of the five academic and four community partners (two from each FQHC) on this CBPR study. The second and senior authors worked on the original CCL study and suggested the process mapping as a collaborative research method. The fifth and sixth authors were involved in integrating CHWs into their clinical services in FQHC 1 and supervised the CHWs.The third author is the research and grants manager at FQHC 2. The fourth author, an experienced CHW, was involved in the CCL study at FQHC 2.The first author is a graduate research associate and PhD student who joined the study based on her previous work as a medical assistant in family medicine and her interest in health research. At the time of the study, the seventh author was an undergraduate public health student who helped to implement the process map with FQHC 1 as part of an independent study.

### FQHC settings

FQHC 1 currently has seven locations serving a metropolitan area of central Arizona and offers adult medicine, pediatrics, behavioral health, crisis intervention, and other social services. FQHC 1 initially provided behavioral health services and expanded to include primary care in recent years. FQHC 1 serves approximately 5000 patients across its locations. FQHC 2 currently has thirteen locations to serve a metropolitan area of southern Arizona and offers adult medicine, behavioral health, dental services, family medicine, pediatrics, and other specialized services, such as addiction treatment and family planning. FQHC 2 serves approximately 128,500 patients across its locations. Both FQHCs are located in major urban areas and deliver primary care services to lower income and historically underserved populations. FQHC 1 began employing CHWs at their organization in 2012, but did not implement formal social risk screening until 2019. FQHC 2 began formally working with CHWs in 2010 and implemented formal social risk screening at their organization in 2018. Both clinics had a history of working with CHWs to conduct community outreach, health promotion initiatives, and address clinical priorities. Therefore, once each clinic began formally screening for social risk, CHWs were a natural fit for this role.

### Process mapping for organizational evaluation

The academic and community partners decided to engage in process mapping as a means to describe strengths and identify barriers to social risk screening and follow-up. Process mapping is a process evaluation tool designed to assist health organizations in evaluating systems-level interventions to improve the quality of health care service delivery [26]. In a systematic review, Antonacci et al. (2021) identified five stages of process mapping that include (1) preparation and planning; (2) gathering data; (3) map generation; (4) map interpretation; and (5) application of results [26]. 

### Phase I: preparation and planning

The community and academic partners collectively met monthly over the course of several months for one hour via Zoom to share the history of social risk screening in their respective organizations, develop study aims, and determine a framework to guide the study. The community partners selected the Consolidated Framework for Implementation Research (CFIR 2009) due to their familiarity with the framework and it’s fit for their clinical context. CFIR helped the partners broadly consider factors internal and external to the clinical settings that needed to be explored. The partners utilized the 2009 version of the framework, although it should be noted that an updated version (2022) does exist [27, 28]. Subsequently, the CBPR team developed questions for the process map evaluation, designed to document the flow of social risk screening, referral, and follow-up across departments in their organizations, including barriers and facilitators to the process.These questions were used at both FQHC’s process mapping activity and were used to prompt and probe the process map development. Question development was designed to identify aspects of the inner and outer context that might influence social risk screening, referral, and follow-up procedures (Table 1) and were based on CFIR domains [27]. The CFIR (2009) domains include: intervention characteristics, outer setting, inner setting, characteristics of individuals involved, and the process of implementation [27]. Definitions for each domain can be found in Table 1.

For the purposes of this study, questions were developed for only three CFIR domains, as decided by our CBPR team: outer setting, inner setting, and the process of implementation. The team concluded that the two excluded CFIR domains were outside the scope of the process map that we aimed to develop for this study.

### Process map guiding questions

**Table 1:** Describes the questions generated by our CBPR team to guide the process mapping activities

CFIR Domain	Questions Generated by CBPR team
*#2: Outer setting* The setting in which the Inner setting exists (i.e.: community where FQHC system resides)	*1.How well do you think that the current social risk screening and referral process meets the needs of the patients and families served by your organization?*
*#3: Inner setting* The setting in which the innovation is implemented (i.e.: FQHC)	*1.What infrastructure changes would you suggest to better screen and make referrals for social risk?*
	*2.Is there support offered during the administration of the social risk screening? (Who can patients ask questions to when filling out the tool? )*
*#5: Process of implementation* The activities and strategies used to implement the innovation.	1.*Describe how you currently screen for social risks in your clinic. Who is involved in administering it? How often do patients receive screening?* * · (For clinicians) Is it a facilitator or barrier for medical decision making?* * · Which staff members implement the social risk screening tool?* * · Who answers the screening questions? Is it a patient or a staff member? What is the age requirement for patients to fill out the social risk screening tool?*
	*2.What facilitates the successful completion of social risk screening/referrals?*
	*3.What are the biggest barriers to screening/referrals?*
	*4.Who follows up after the social risk screening is completed? What support is given? How does the patient expect to be contacted?*
	*5.What adaptations have you made to your social risk screening process? Why?*

Additionally, each community partner took the lead in determining who from their staff should participate in the data gathering phase, determining ideal times for the activity, and inviting participation of appropriate organizational members from the respective FQHC. Process map participants were invited across all locations for each FQHC. Process map participants from FQHC 1 included: a site supervisor, chief operating advisor, director of operations, regional supervisor, clinical supervisor, clinical director, peer support supervisor, and two CHWs. In contrast, FQHC 2 made an internal decision to divide the process map evaluation into two sessions: the first with CHWs and a CHW supervisor only; the second with clinical staff only. Process map participants for FQHC 2 included: one CHW supervisor and four CHWs. Subsequent process mapping at our second partnered FQHC included: one dentist, one internal medicine physician, and a research and grants manager.

### Phase II: gathering data

The academic partner took the lead in facilitating the process map evaluation activity separately at each FQHC. The process mapping exercise took place over the employees’ lunch hour with food and refreshments provided by the academic partner team. Using the CFIR questions developed by the CBPR team to guide conversation, one academic team member drew the workflow and various pathways of the organization’s procedures for social risk screening, referral, and follow-up efforts on a whiteboard, making note of the context and situations in which these procedures might occur. The other academic team member(s) took careful notes of the actual responses to the questions in order to capture contextual factors to the map and the participants’ insights and perspectives. The notes taken during the process mapping activity were used to identify common barriers to social risk screening, referral, and follow-up procedures across both FQHCs. This process was not recorded or transcribed.

### Phase III: map generation

Members of the academic partner team created a visual diagram of the process maps for each FQHC with Lucid.app software. The visual diagram represented the various pathways of the organization’s procedures for social risk screening, referral, and follow-up efforts by using different colors and symbols. The academic partners then delivered the process map of each organization’s workflow to each partnering FQHC.

### Phase IV & V: map interpretation & application of results

**Fig. 1:**
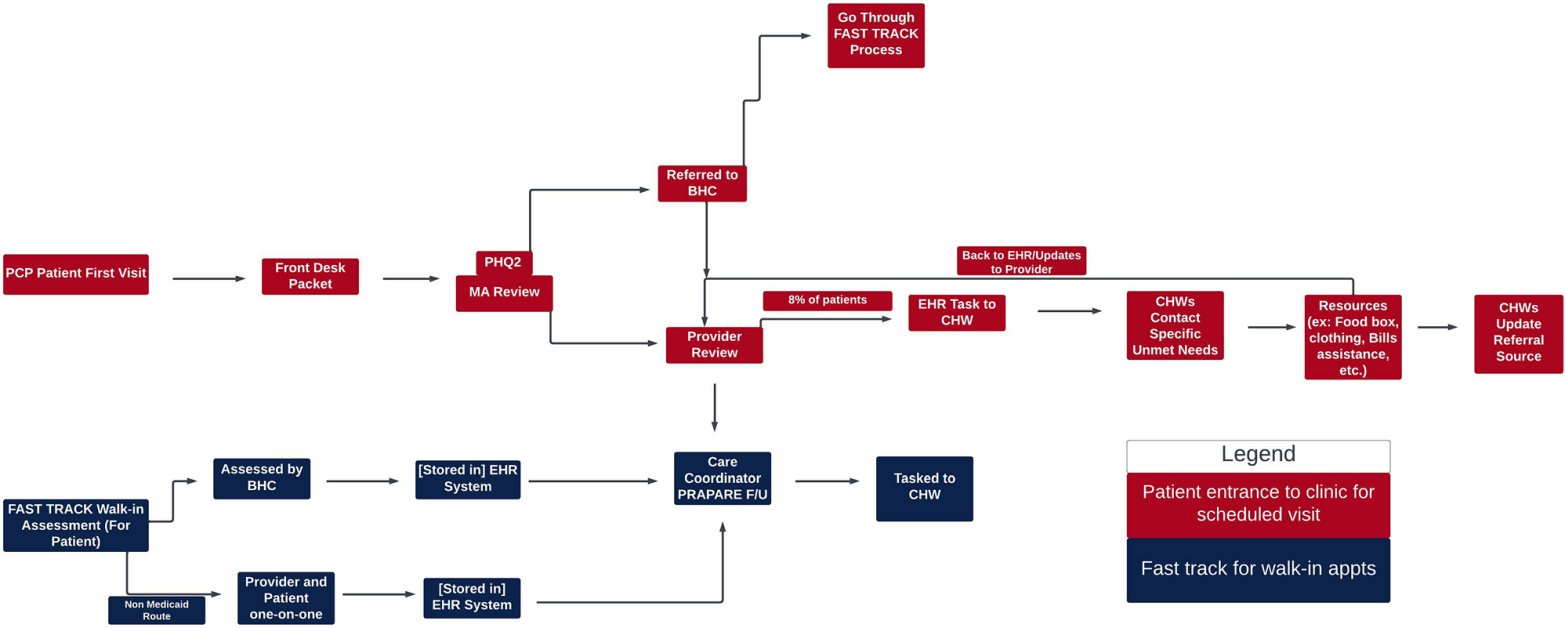
Depicts the process map generated by FQHC 1

**Fig. 2:**
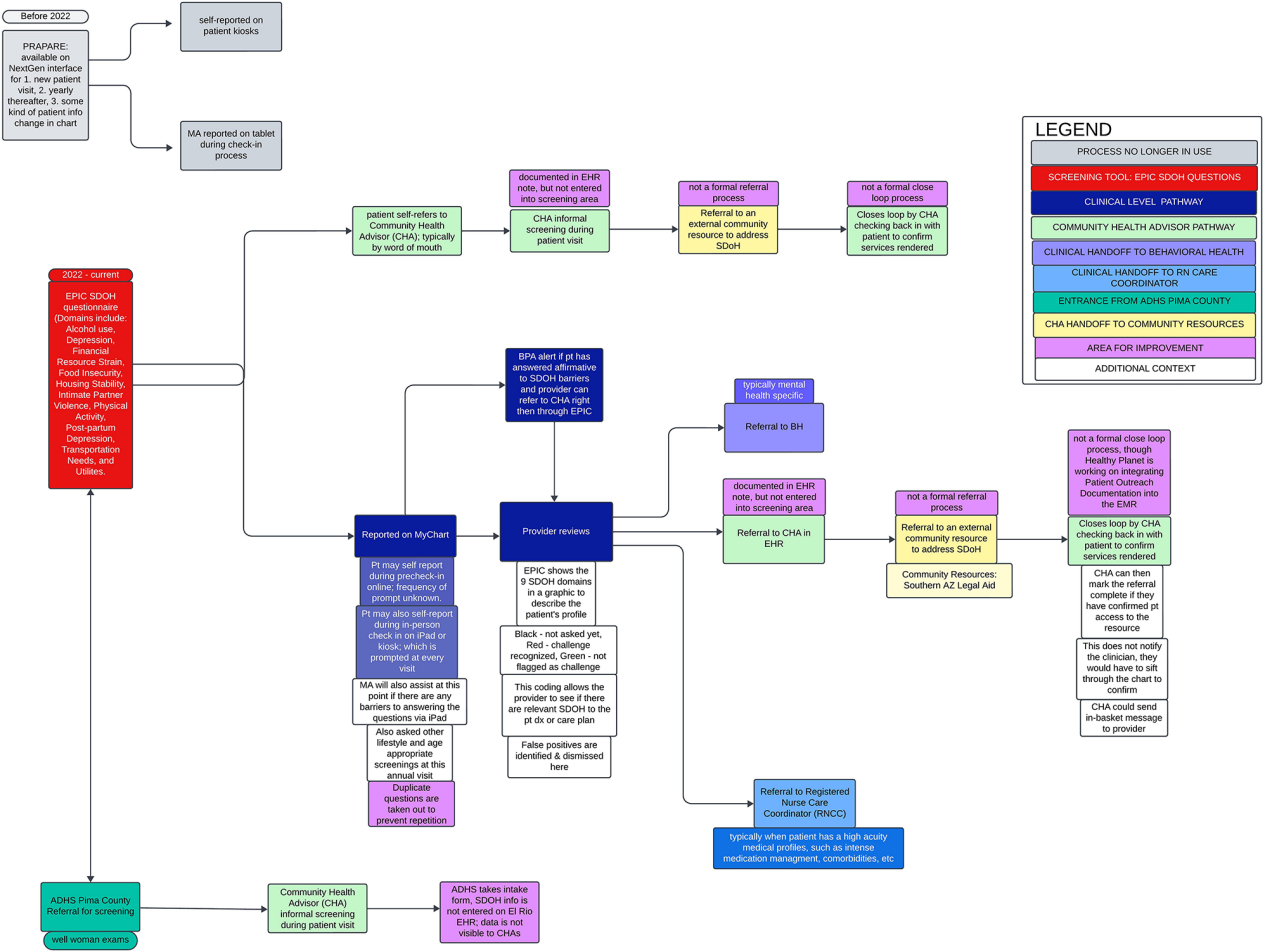
Depicts the process map generated by FQHC 2

Academic partners met with each partnering FQHC individually via Zoom for one hour, within the following month after the process mapping occurred, to make adjustments and clarify any map inaccuracies. During this time, academic and community partners also met to discuss the themes extrapolated from the process mapping notes. Final process map products were delivered to each FQHC partner for their internal use in Phase V of the study, which included organizational system improvements to address inefficiencies and opportunities for improvement identified during the process evaluation (Fig. 1 and 2).

## Results

The process evaluations at our partnering FQHCs resulted in two process maps of each organizations’ workflow of social risk screening, referral, and follow-up. The generated process maps also focused on the integral role of CHWs from screening to follow-up of social risks. The process maps for FQHC 1 and FQHC 2 are found in Fig. 1 and 2, respectively. It is of note that each process map is an individual representation of the FQHC. Further, each FQHC opted to create their process map based on their needs and included their identified participants to fit their clinical context. Thus, the two process maps differed in format as they reflected the unique operations and needs of each clinic. In addition to the generated process maps for each partnering FQHC, the academic partners identified barriers to the social risk screening, referral, and follow-up processes. These findings are grouped generally by CFIR domain and described in Table 2.

**Table 2:** Common identified barriers for future areas of quality improvement between FQHC 1 and FQHC 2

*CFIR Domain #2: Outer setting*	1.* Accessing Resources: Not enough resources for complex social risks*
*CFIR Domain #3: Inner setting*	1. *Lack of Standard Entry Points: Entry points (across departments) are not consistently screening for social risks across the organization* 2. *Lack of Standard Screening Policy: No standard policy across the organization for how often or how screening takes place each time a patient is seen* 3. *Variation in Provider Understanding of CHWs: Too much variation in healthcare provider knowledge of CHWs and their role*
*CFIR Domain #5: Process of implementation*	1. *IT Challenges: Changes to different EHR system disrupting momentum on social risk screening procedures* 2. *Screening Tool Changes: Changes to the screening tool used at the organization over time resulting in inconsistent procedures and confusion* 3. *Lack of Communication: Lack of communication between CHWs and healthcare providers in the social risk screening*,* referral*,* and follow up process* 4. *Use of Social Risk Data Concerns: Concerns about how social risk data will be used and how useful it is for the FQHC staff and patients* 5. *Administrative Burden: Administrative burden of completing the tool and following up on referrals*

### Process maps Figs. 1 and 2

## Discussion

Along with the creation of two unique process maps that provided each FQHC with a visual representation of how patients are assessed and treated for their social needs, the academic partner team identified common barriers to social risk procedures for the three CFIR domains utilized in this study: outer setting, inner setting, and process of implementation. The themes and contextual information were extracted from process map activity notes and were directly related to the resources required for adequate CHW integration for social risk diagnostics and treatment in the primary care setting.

### Outer setting: accessing resources

The understanding of social risk screening, referral, and follow-up within the organization did not change CHW perspectives on the promise that social risk identification can have on meeting patient needs in their community. There appeared to be consensus that when social risk screening was completed by the CHW there was generally not an issue addressing the identified need. However, CHWs acknowledged the struggle to secure community resources, especially for complex social risks. For example, the CHWs portrayed a confidence that if issues around nutrition were identified, they could locate community resources to alleviate that social pressure. However, for social risks such as homelessness, locating resources for those already experiencing homelessness proves harder than locating resources for those at risk of homelessness due to lack of community resources. This discussion further confirmed the necessity of early and consistent use of social risk screening throughout the organization.

### Inner setting: Lack of standard entry points, lack of standard screening policy, and variation in provider understanding of CHWs

The staff involved in the process mapping reflected on the patient entry points for social risk screening at their organization. There was an emphasis on the need to cast a greater net for social risk screening upon entrance to any clinic service provided at the FQHC. Similarly, FQHC staff highlighted that leadership awareness of areas that are lacking efficient social risk screening, referral, and follow-up is necessary. For example, at one of the partnering FQHCs, a dental clinician was unaware of the scope of CHWs’ work and how they can be relied on as a resource at their organization to support patients. Therefore, there was a call from FQHC staff for a formal way for clinicians to refer patients to CHWs, document community resources available, and centralize community referrals for increased follow-up. In that respect, there was also a discussion on the importance of considering the lost-to-follow-up for patients who do not recurrently receive services at the FQHC and how to best serve this dynamic population. FQHC staff noted the importance of capturing the needs and utilization of services by the most vulnerable patients, which starts with streamlining efforts between clinical staff, from dental to primary care, with CHWs to effectively screen for and address social risks.

### Process of implementation: IT challenges, screening tool changes, lack of communication, use of social risk data concerns, and administrative burden

Across the iterative rounds of process mapping, IT challenges in the EHR were consistently cited as barriers to implementation of social risk screening and responsive actions as a policy standard. More specifically, changes in the type of EHR was one cause of a fragmented method of social risk screening at FQHC 2. CHWs expressed that the previous EHR system their organization used was more comprehensive in capturing social risk information. Thus, with a change to their new system, the previous social risk screening protocol was discontinued, leading to CHW dissatisfaction and concerns that there was lower reach for social risk screening and treatment in their community. Similarly, CHWs lacked a comprehensive understanding of the policies of other departments in routine screening and next steps after social risk was identified. Further, CHWs confirmed that there was limited communication between their team and providers or clinical staff after CHWs received an internal patient referral for an identified social risk.

Additionally, across CHWs and clinical teams, there was concern over the usefulness of the EHR to document the social risk if there was no standard plan on how the data would inform organizational policy. That is, without a clear procedure for action once social risk is identified, the administrative time necessary to use a social risk survey tool in the EHR may not serve the patient, CHW, or the clinical provider, which may undermine the understanding and importance of using this assessment in a standardized manner. However, CHWs and clinical staff recognized the importance of documenting issues of social risk to provide continuum of care to patients. Both expressed a desire to act on the identified social risks with the belief that this action can make a difference in patients’ lives.

### Impact of process mapping on partnering FQHCs

This process evaluation study on process mapping of social risk identification and intervention prompts a discussion around the role of CHWs in screening, referral, and follow up, and the resources required for improvements to the current system. More specifically, both process maps highlight key methods to improve addressing social risk within FQHCs. This process map evaluation revealed that CHWs should be integral members of the primary care teams, with respect to their ability to identify barriers to current processes for social risk screening, referral, and follow up. The process evaluation, which relied heavily on the expertise of CHWs, has been shared with upper-level FQHC leadership so that action may be taken to expand the patient population reached and improve on ways to address the identified social risks of patients. Since completion of the process mapping, one of the partnering FQHCs has used the results to investigate their internal processes and make operational modifications, such as locating CHWs into their primary care offices for greater ease of communication with clinicians on patients’ social risks and needs.

It is also worth noting that it was important that community partners led the process map activity conceptualization and implementation through CBPR principles, as each organization has a unique workflow and different insight into their processes. This uniqueness is captured in Fig. 1 and 2, as they vary drastically in their representation of internal processes. The community partners on the research team were invested in the success and accuracy of the evaluation with the goal of improving patient care. Without a CBPR approach, our team likely would not have captured the variations in clinic operations to understand current procedures around social risk screening, referral, and follow up. Further, process mapping has given our partnering FQHCs the ability to assess their internal systems, identify weaknesses and opportunities for improvement, and strengthen the role of CHWs in their primary care teams.

### Limitations

There are several limitations related to this process evaluation. First, while the study aims to understand the role of CHWs in screening, referral, and follow-up of social risks, the process evaluation was only done at two FQHCs in Arizona. Therefore, this study does not seek to be generalizable in its findings. This study was based on the experiences within FQHCs, which have a unique role in providing care to underserved populations. This approach may be useful for other care models, such as integrated health systems, but process mapping would need to be repeated in this setting. Additionally, participants for the process mapping activity were self-selected or were selected by community partners of the same organization to participate. This should be noted as a potential limitation, as the data gathered reflects those most likely to be involved in social risk screening, referral, and follow-up procedures to begin with, thereby potentially skewing the organizational perspective. However, as a process evaluation, it was our goal to reflect upon potential areas for quality improvement for CHWs integration into primary care and improvement in social risk identification and intervention. Therefore, while our team made efforts to hear the voices of diverse FQHC staff, this was shaped by organizational culture and decision-making.

Further, the scope of this study did not include surveying each FQHCs’ patient population to understand the patient perception of social risk screening, referral, and follow-up. Similarly, this study did not extend past the partnering FQHC to community-based organizations to garner information on how to feasibly and effectively establish channels for following up or “closing the loop”. Future research should extend the process evaluation to the collaborative work between referring CHWs and community-based organizations.

Lastly, our CBPR team used an older version of CFIR (2009) because of the team’s lack of awareness of the 2022 version at the time of study conceptualization. However, we simply used CFIR as a guiding framework in developing questions for process mapping and contextualizing information. Future research and efforts to replicate should see the 2022 version of CFIR [28]. 

## Conclusions

The social risk process evaluation at two of Arizona’s major urban FQHCs revealed key opportunities to improve the reach and effectiveness of social risk screening and intervention for some of the most vulnerable populations in healthcare. The results of this process evaluation further support the critical role that CHWs can have, as integral members of primary care teams, in improving the social risk screening, referral, and follow-up procedures. Findings underscore the importance of collaborative primary care teams to streamline social risk screening, referral, and follow-up within healthcare delivery organizations. Integration of CHW efforts in primary care teams to address social risk, with clear processes for social risk screening, referral, and follow-up, may improve communication across teams and provide a method to introduce social prescription into medical decision-making for clinicians. More work must be done to further document the efficacy and effectiveness of CHW integration into primary care teams in order to incorporate social prescription into the healthcare delivery model. Our CBPR team aims to use the initial FQHC process maps of this study to develop a statewide survey in which Arizona FQHCs will document their own organizational processes for social risk screening, referral, and follow-up. This data will be used to propose models for CHW integration into primary care in the FQHC setting for a centralized social risk screening tool and an accessible community resource referral system.

## Electronic supplementary material

Below is the link to the electronic supplementary material.

Supplementary Material 1

## Data Availability

All data generated or analyzed during this study are included in this published article.
