# Supplementary material for: Centering the role of community health workers in social risk screening, referral, and follow-up within the primary care setting
Source: BMC Prim Care. 2024 Sep 13;25:338. doi: 10.1186/s12875-024-02590-3 (PMC11396075; doi:10.1186/s12875-024-02590-3)
Supplement: Supplementary file 1 — Supplementary Material 1 [file 12875_2024_2590_MOESM1_ESM.docx]

*Process Map Guiding Questions*

| ***CFIR Domain*** | ***Questions Generated*** |
| --- | --- |
| *#2: Outer setting* | *1.* *How well do you think that the current SDOH screening and referral process meets the needs of the patients and families served by your organization?* |
| *#3: Inner setting* | *1.* *What infrastructure changes would you suggest to better screen and make referrals for SDOH?* |
|  | *2.* *Is there support offered during the administration of the social risk screening? (Who can patients ask questions to when filling out the tool?)* |
| *#5: Process of implementation* | *1.* *Describe how you currently screen for SDOH in your clinic. Who is involved in administering it? How often do patients receive screening?*  *·* *(For clinicians) Is it a facilitator or barrier for medical decision making?*  *·* *Which staff members implement the SDOH screening tool?*  *·* *Who answers the screening questions? Is it a patient or a staff member? What is the age requirement for patients to fill out the SDOH screening tool?* |
|  | *2.* *What facilitates the successful completion of SDOH screening/referrals?* |
|  | *3.* *What are the biggest barriers to screening/referrals?* |
|  | *4.* *Who follows up after the SDOH screening is completed? What support is given? How does the patient expect to be contacted?* |
|  | *5.* *What adaptations have you made to your SDOH screening process? Why?* |
